# Quantum-Indeterminate Proton Positions in Ultrafast Excited-State Intramolecular Proton Transfer

**DOI:** 10.1021/acs.jpclett.6c01188

**Published:** 2026-04-29

**Authors:** Minhyuk Lee, Changmin Lee, JunWoo Kim

**Affiliations:** † Department of Chemistry, 34933 Chungbuk National University, Cheongju 28644, Republic of Korea; ‡ Department of Chemistry, 3270 Incheon National University, Incheon 22012, Republic of Korea

## Abstract

Although molecular vibrations are fundamentally described within a quantum mechanical framework, nuclear motion in condensed-phase chemistry is typically treated semiclassically, because direct experimental signatures of quantum behavior are difficult to access. Excited-state intramolecular proton transfer (ESIPT), which occurs before significant intra- and intermolecular perturbations can influence the system, provides a unique opportunity to probe such regimes. Here we investigate the ESIPT dynamics of [2,2′-bipyridyl]-3,3′-diol (BP­(OH)_2_), a system involving the transfer of two protons, using coherent vibrational spectroscopy. In a semiclassical picture, proton transfer is expected to impulsively drive coherent vibrations of the molecular framework through momentum conservation. In contrast to this expectation, only one of the two proton transfer events exhibits such a proton-transfer-induced coherent vibration, while the other shows no evidence of a coherent response associated with proton transfer. This observation cannot be straightforwardly explained within a simple semiclassical description of sequential proton transfer. Our results demonstrate how coherent vibrational spectroscopy can reveal subtle quantum aspects of nuclear motion in ultrafast proton-transfer reactions.

## Graphical abstract

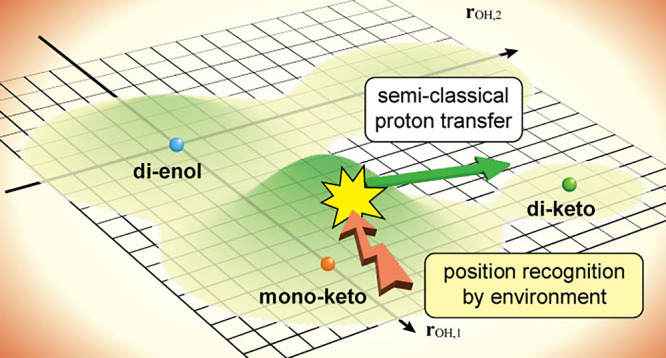

Understanding molecular vibrations has long provided a foundation for exploring the structure and dynamics of matter, particularly in gas-phase systems where well-isolated molecules exhibit discrete vibronic and rovibrational transitions governed by quantum mechanics. In such regimes, vibrational motion is characterized by well-defined quantum numbers, yielding precise momentum information while rendering the molecular position inherently uncertain under the Heisenberg principle. By contrast, molecules in condensed phases experience strong interactions with their surrounding environment, which increase the number of accessible degrees of freedom. As a result, nuclear motion in the condensed phase is often well described using semiclassical approximations.^1–4^ In this context, a *classical* description refers to a representation in which the nuclear position is treated as localized and follows a well-defined path on the potential energy surface. Nevertheless, high-frequency vibrational modes can still show quantum behavior, since their energy far exceeds the effects of thermal fluctuations and environmental broadening, leading to signals that cannot be explained by a purely classical picture.^5–8^

Ultrafast excited-state intramolecular proton transfer (ESIPT) provides an interesting platform for examining the boundary between quantum and classical descriptions of nuclear motion. Molecules undergoing ESIPT typically exist in the enol form prior to photoexcitation and convert to the keto form upon excitation.^9–11^ In the enol configuration, the O–H stretching mode constitutes a major component of the proton-transfer coordinate, simultaneously increasing the distance to the donor and decreasing the distance to the acceptor. Because this mode typically exhibits a high frequency on the order of 3000 cm^–1^, ESIPT can be expected to exhibit quantum mechanical behavior.^12^ However, coherent vibrational spectroscopy, which provides direct information on photoinitiated nuclear motion, shows that many ultrafast ESIPT processes can be described within a semiclassical framework.^13,14^

Recent femtosecond time-resolved studies have reported partial quantum signatures in ultrafast ESIPT, indicating that proton motion in these systems does not always follow a purely classical trajectory.^15,16^ When the ESIPT can be described semiclassically, low-frequency vibrational modes typically play a dominant role in the dynamics. In contrast, when high-frequency modes such as the O–H stretch constitute a major component of the reaction coordinate, a classical description based on a well-defined proton position becomes inadequate. Demonstrating quantum behavior, specifically the indeterminacy of the proton position at the moment of ESIPT, would require direct access to the transient proton configuration immediately following excitation. However, such information is inherently difficult to obtain, as it remains unclear whether the absence of a well-defined position reflects a fundamental indeterminacy or a limitation of the measurement.^17–19^

When direct access to the instantaneous proton position is not available, analyzing the molecular dynamics induced by the ESIPT provides an alternative route to infer its behavior. Within a semiclassical framework, the proton is assumed to possess a well-defined position and momentum, and ESIPT proceeds as a localized transfer event. In this case, the interaction between the proton and the acceptor is expected to impulsively drive coherent vibrations of the molecular framework (Figure 1a). In contrast, if the proton’s vibrational wave function remains close to an eigenstate, as illustrated in Figure 1b, the proton transfer does not generate additional coherent vibrational motion. Instead, the proton wave function couples to the vibrational degrees of freedom of the keto form and evolves into eigenstates of the product configuration.^20^ As a result, the formation of the keto state is identified through its characteristic spectral response.

**Figure 1:**
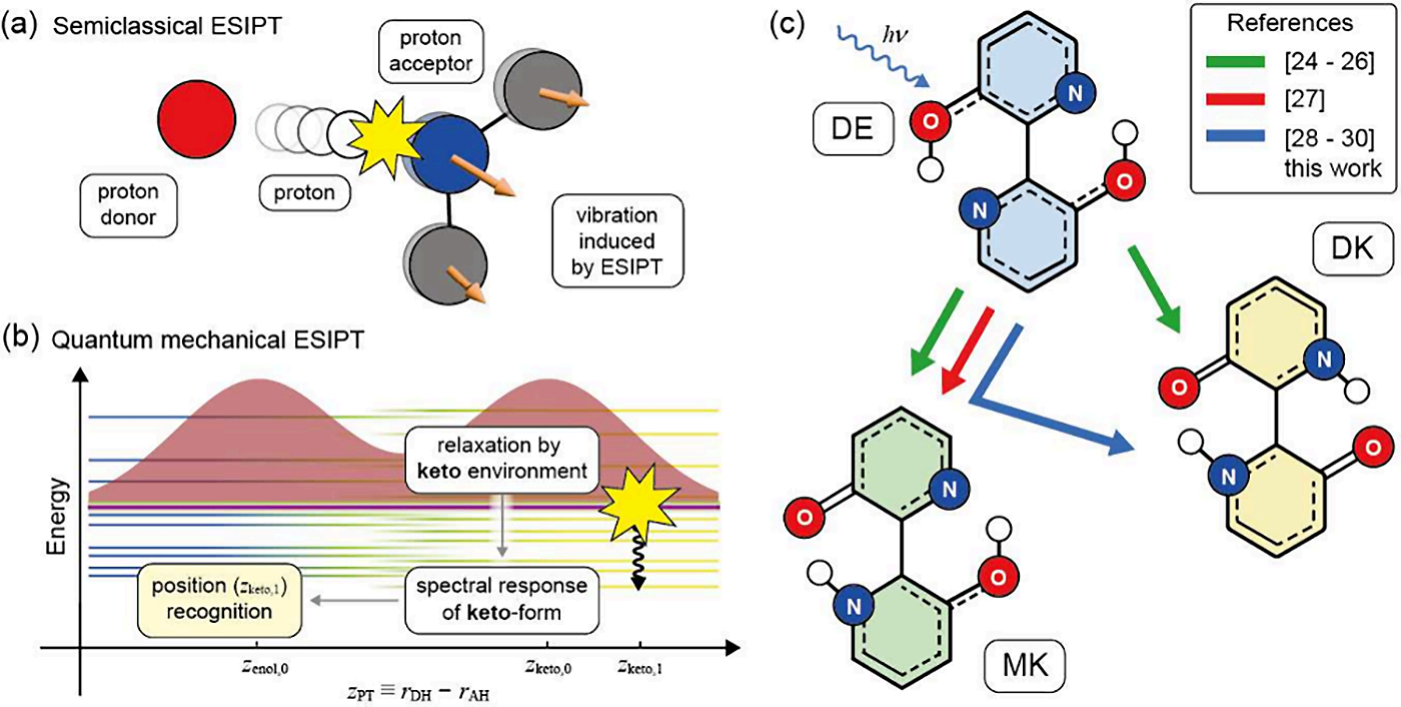
(a) Schematic illustration of ESIPT dynamics in a semiclassical framework. (b) Vibrational wave function describing a proton between proton donor and acceptor on the energy diagram associated with the enol- (**z**_*enol*,0_) and keto-forms (**z**_*keto*,0_). (c) Excited-state dynamics of BP­(OH)_2_ occurring on an ultrafast time scale (<300 fs).

Forming a vibrational wave function that is delocalized between the enol and keto wells immediately after photoexcitation requires that the proton and the acceptor be positioned sufficiently close in the enol geometry. Molecules such as [2,2′-bipyridyl]-3,3′-diol (BP­(OH)_2_) (Figure 1c) or 10-hydroxybenzo­[*h*]­quinoline (HBQ) satisfy this structural condition because the donor, proton, and acceptor lie on a six-membered ring, allowing the enol form to remain stable while still maintaining a short proton–acceptor distance. Previous studies on HBQ have shown that transient shortening of the donor–acceptor distance, driven by skeletal motion, increases the probability of proton transfer.^14,15^ Although the stochastic nature of the transfer reflects a quantum aspect of the proton coordinate, the coherent vibration induced by the proton transfer is usually interpreted within a semiclassical framework.

BP­(OH)_2_ is more flexible than HBQ, and solvent fluctuations can readily impose asymmetry between the two O–H···N motifs.^21,22^ As a result, at the moment of photoexcitation, one of the protons is likely to be positioned closer to its acceptor than the other. This structural bias increases the probability that the vibrational wave function associated with the symmetric or asymmetric O–H stretching modes of the reactant (DE*) retains its quantum character, making BP­(OH)_2_ a favorable system for observing quantum signatures of proton motion. This possibility has important implications for interpreting earlier work, because the excited-state dynamics of BP­(OH)_2_ have remained unresolved for decades (Figure 1c).^23–30^ Early femtosecond studies performed before 2010 faced technical limitations, particularly in time resolution and signal-to-noise ratio, which made it difficult to distinguish the relevant dynamical components. It is also possible that the absence of a clear consensus may stem from the fact that quantum aspects of the proton coordinate were not explicitly considered in earlier interpretations.

In this study, we investigate the excited-state dynamics of BP­(OH)_2_ to uncover quantum-mechanical behavior associated with proton motion, using coherent vibrational spectroscopy.^31–33^ By generating near-UV pulses with a duration of approximately 15 fs and implementing a rapid-scan, frequency-modulated detection scheme for transient absorption (TA) measurements, we were able to measure coherent vibrational wavepackets with high signal-to-noise ratio over a broad spectral range (see Supporting Information Section 1 for details on the TA measurement). A striking feature of the data is that one of the two protons shows no coherent vibrational signature associated with proton transfer, even though the molecule appears to have reached a double-proton-transferred configuration. This observation suggests that one proton is already localized in a keto-like position at the moment of excitation, a scenario that cannot be explained within a classical description and instead points to an intrinsic quantum aspect of the proton coordinate.

Figure 2a shows the TA spectrum of BP­(OH)_2_ dissolved in cyclohexane. Protic solvents were not considered in this study because hydrogen-bonding interactions can directly influence the proton-transfer process.^34,35^ A representative polar aprotic solvent, acetonitrile, was also examined. Although the overall spectral features suggest that the proton-transfer mechanism is similar to that in cyclohexane, significantly faster vibrational dephasing in acetonitrile makes it unsuitable for detailed wavepacket analysis. Therefore, we focus on cyclohexane for the present study (see Supporting Information Section 2 for measurements of BP­(OH)_2_ in acetonitrile). An excited-state absorption (ESA) peak appears in the short-wavelength region (380–480 nm), whereas a stimulated emission (SE) peak is observed in the long-wavelength region (490–650 nm). Because the SE intensity is much weaker than the ESA intensity, the long-wavelength region is scaled by a factor of 10 for clarity.

**Figure 2:**
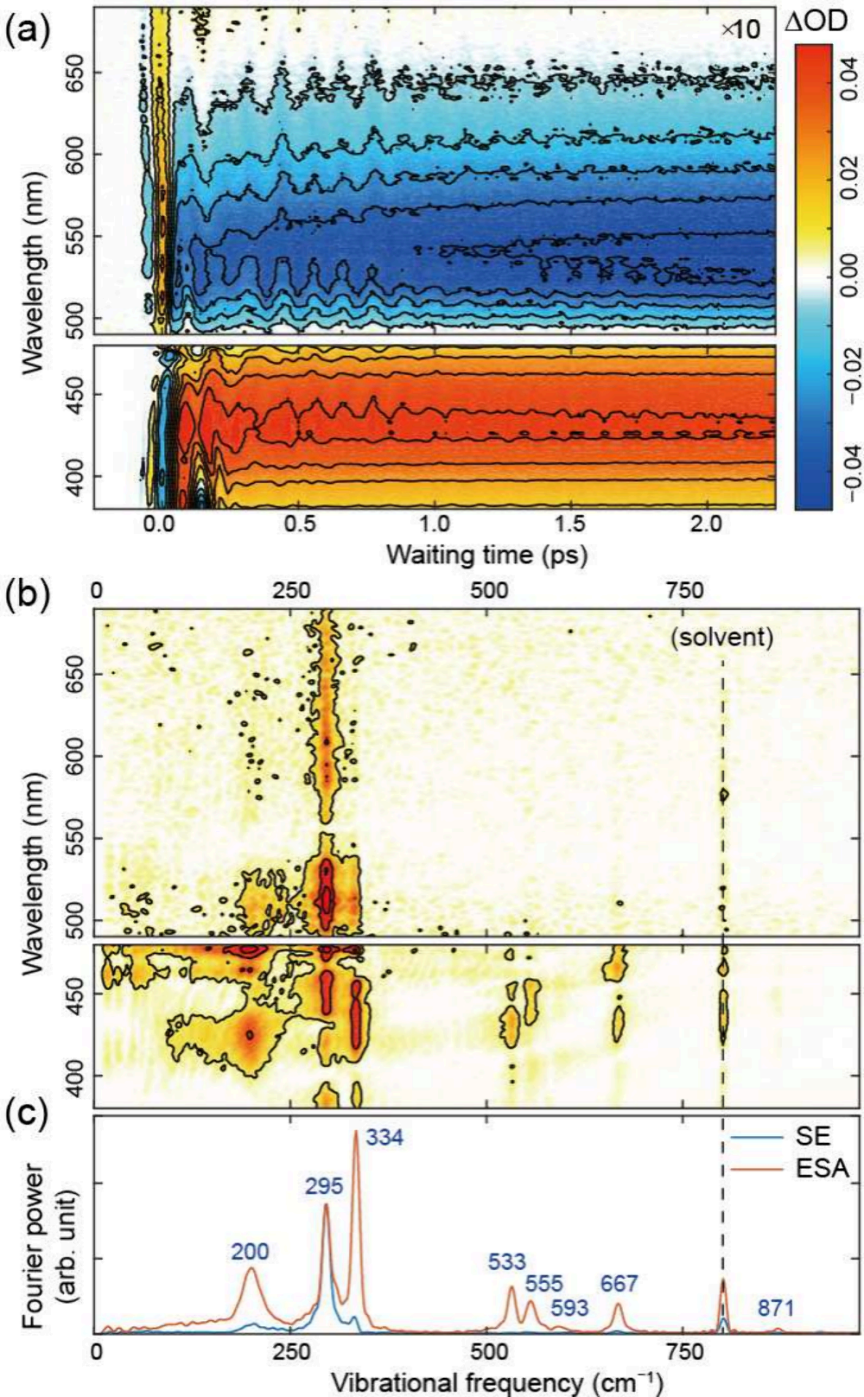
(a) TA spectrum of BP­(OH)_2_ dissolved in cyclohexane. Positive and negative signals are colored red and blue, respectively. The amplitude in the long-wavelength region is multiplied by 10 for clarity. (b) Coherent vibrational map obtained from the TA data in panel a. (c) Coherent vibrational spectra extracted from the red side of the SE band (blue) and the blue side of the ESA band (orange).

Near time zero, a strong SE feature transiently emerges around 400 nm, clearly indicating the population of the excited enol species (denoted as DE*). However, in this early time window, contributions from coherent artifacts make it difficult to extract quantitative decay dynamics for DE*. Coherent artifacts refer to signals arising from nonresonant or undesired light–matter interaction pathways and typically appear as complex features around time zero that are difficult to interpret.^36^ The term *coherent artifact* is often used broadly to encompass nonresonant solvent responses, particularly under strong excitation conditions.

Analysis of the wavepacket oscillations in the TA spectrum enables us to determine whether the SE and ESA peaks observed in the picosecond region originate from a single excited-state species or two distinct species. The oscillations arise from the vibrational wavepacket formed either upon photoexcitation through vibronic coupling^37^ or during the subsequent ultrafast proton transfer,^15^ while their frequencies are determined by the vibrational modes of the final photoproduct. TD-DFT calculations indicate that the excited monoketo (MK*) and excited diketo (DK*) forms of BP­(OH)_2_ exhibit similar nuclear motions but possess noticeably different vibrational frequencies (see Supporting Information Section 3 for details on the quantum chemical calculations). Therefore, if SE and ESA signals arise from different species, their corresponding wavepacket frequencies should differ as well.

Figure 2b shows the 2D coherent vibrational spectrum (CVS) extracted from the TA data, plotted as a function of detection wavelength and vibrational frequency. The peak near 800 cm^–1^ originates from the solvent Raman response and will not be discussed further. Because ESA and SE overlap near 500 nm, the most reliable comparison of their vibrational signatures is obtained by integrating CVS on the blue side of the ESA band and on the red side of the SE band, as shown in Figure 2c. For improved visibility and more direct comparison with the simulations, the Fourier amplitudes were corrected by applying a factor that accounts for the amplitude reduction due to the finite temporal resolution (see Supporting Information Section 4 for the uncorrected data).^38^

When structural evolution occurs on time scales slower than the ultrafast process of interest, yet within the vibrational coherence time, the resulting changes can be reflected in the coherent vibrational wavepacket. Such effects can be examined by evaluating the Fourier-transformed spectra as a function of the time window or by applying time-resolved analysis methods.^39^ In the present system, however, no significant dependence on the Fourier time window is observed (see Supporting Information Section 4). Only the rapidly dephasing vibrational peaks are suppressed at longer delay times, while the remaining spectral features are preserved. This result is consistent with previous studies showing that, after the ultrafast proton transfer, no substantial structural evolution occurs on the sub-10 ps time scale.

Since different electronic transitions exhibit different vibronic coupling strengths, comparing wavepacket amplitudes is not meaningful. Instead, the positions of the CVS peaks provide the decisive criterion. All peak positions in the two integrated CVS traces coincide within experimental uncertainty, indicating that both the ESA and SE features are attributed to the same excited-state species, which is assigned to DK* based on the analysis presented below. The CVS observed in the photoproduct is governed by both the structural differences between the reactant and product states and the mechanistic pathway by which the proton transfer proceeds.^15,40,41^ By combining ultrafast measurements with quantum-chemical calculations of the ground state (GS), MK*, and DK* geometries and by identifying vibrational modes that are activated by proton transfer, we can determine which of the two species is predominantly formed at early times.

The structural differences between the reactant and product can be expressed as displacements along the normal modes using the normal-mode projection method.^14,42^ For each mode, the product of the squared displacement and the vibrational frequency yields the vibrational reorganization energy, which is proportional to the amplitude of the corresponding wavepacket oscillation. This approach is applicable when the two structures involved in the projection differ only modestly. However, proton transfer accompanies a substantial change in the proton’s position, which violates this assumption. To account for this, we separately considered wavepacket contributions arising directly from the proton-transfer coordinate and those arising from the remaining structural differences unrelated to proton motion. The resulting theoretical CVS is shown in Figures 3a and 3b (see Supporting Information 5 for details).

**Figure 3:**
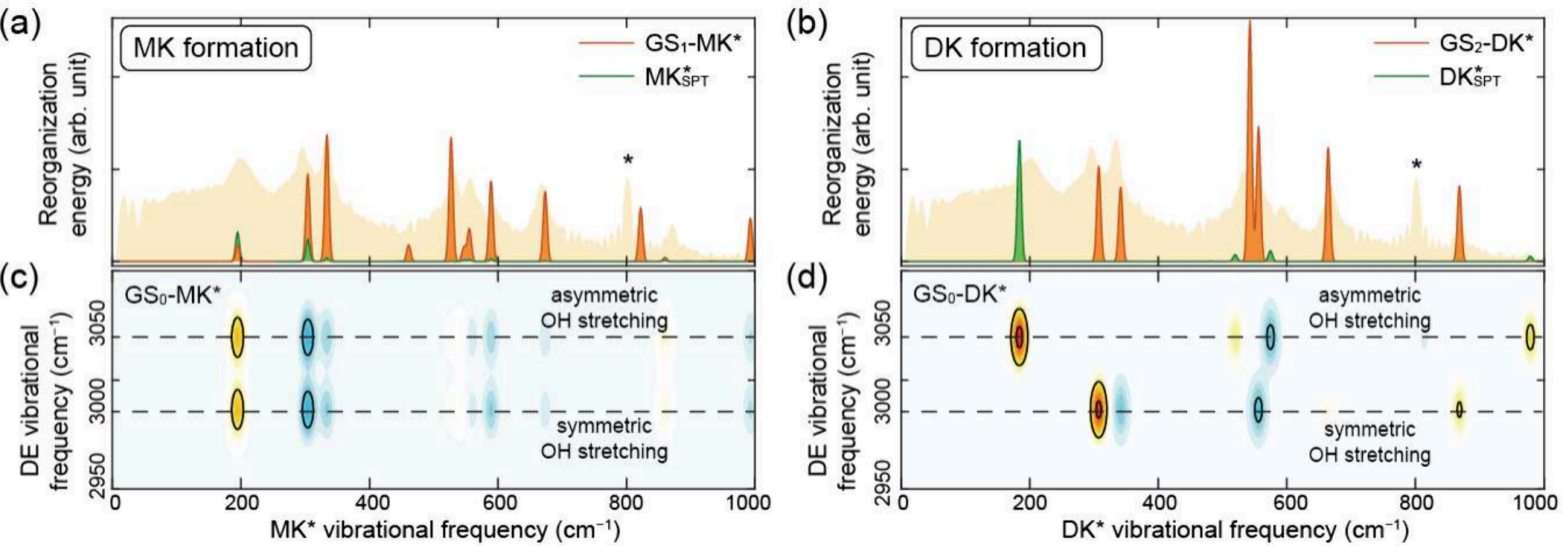
(a-b) Theoretically predicted CVS based on the structural difference between single-proton-transferred GS (GS_1_) and MK* (panel a) and between double-proton-transferred GS (GS_2_) and DK* (panel b) (orange). Green lines represent simulated coherent vibrational peaks attributed to single proton transfer (SPT), constructed from the corresponding Duschinsky matrices with an arbitrary scaling factor. (c,d) Duschinsky matrices representing the correlation between the vibrational modes of GS and MK* (panel c) and between GS and DK* (panel d).

The experimentally measured CVS (Figure 2c) shows clear coherent vibrational peaks near 200, 295, 334, 533, 555, 593, 667, and 871 cm^–1^. Comparison between the experimental CVS and the simulated spectra indicates that the contribution arising from backbone structural differences (orange lines in Figures 3a and b) is more pronounced than that arising from the proton-transfer coordinate (green lines), so we first examine the backbone-driven contributions. For the model in which MK* is assumed to be the initial photoproduct (Figure 3a), most of the experimentally observed peaks are reproduced. However, several predicted peaks (460, 820, and 992 cm^–1^) are not observed experimentally, and the calculated intensity of the 871 cm^–1^ mode is significantly weaker than the measured one. In contrast, the model in which DK* is the final product (Figure 3b) shows better agreement with the experimental data.

In the GS, the proton motion is primarily described by the symmetric and asymmetric stretching modes. Consequently, vibrational modes generated in the photoproduct through impulsive proton motion are expected to couple strongly to these two stretches. The extent of this coupling can be estimated using the Duschinsky matrix, which quantifies the correlation between the normal modes of the GS and those of the photoproduct^42^ (see Supporting Information Section 6.1 for details on Duschinsky matrix). For MK*, the Duschinsky analysis shows that both O–H stretching modes in the GS contribute nearly equally to all vibrational modes below 1000 cm^–1^ (Figure 3c). The asymmetric stretch corresponds to motion in which one proton approaches its acceptor more rapidly than the other and is therefore strongly coupled to single-proton transfer, whereas the symmetric stretch describes motion associated with simultaneous double proton transfer. The coherent vibration arising from the single-proton-transfer pathway is shown as a green trace in Figure 3a. Because the vibrational displacements induced by proton transfer and those associated with backbone structural displacements have the same sign (see Supporting Information Section 6.2 for additional simulation data), destructive interference between the two pathways is unlikely, and no additional cancellation of coherent vibrational signals must be considered.

The vibrational modes of DK* exhibit a markedly different correlation with the symmetric and asymmetric stretching modes compared with those of MK*, as shown in Figure 3d. Notably, even when the contribution from the symmetric O–H stretch, which corresponds to simultaneous double proton transfer, is included, the DK* simulation does not reproduce the 200 and 593 cm^–1^ peaks. In contrast, the asymmetric O–H stretch, which represents single proton transfer, activates both of these modes, as indicated by the green lines in Figure 3b. The observation of the 200 and 593 cm^–1^ peaks therefore supports the involvement of sequential proton transfer. However, note that this observation does not necessarily exclude the presence of a concerted pathway. Because BP­(OH)_2_ is structurally flexible, significant structural inhomogeneity is expected in solution. In addition, the CVS exhibit a strong dependence on excitation frequency,^26^ suggesting that the reaction dynamics may involve heterogeneous pathways rather than a single well-defined mechanism. If DK* is formed through a concerted double-proton-transfer pathway, the resulting coherent vibrational wavepacket, corresponding to the symmetric O–H stretching coordinate, would likely overlap spectroscopically with peaks arising from backbone structural deformation (orange area in Figure 3b). As a result, it is difficult to extract detailed information about a concerted pathway from the present measurements. In the following discussion, we therefore focus on the coherent vibrational peaks generated by sequential proton transfer to elucidate the detailed dynamics of this process.

If the two protons are transferred sequentially, then each proton transfer should leave a distinct vibrational signature, meaning that each transfer should generate vibrational coherence in the low-frequency modes that are coupled to the corresponding proton transfer. This expectation follows from semiclassical considerations in which momentum associated with proton displacement must be conserved, a framework that has successfully explained vibrational coherence in the ESIPT of HBQ.^15^ In a two-step semiclassical proton transfer, the first proton transfer would generate low-frequency coherence in the MK* intermediate, and once the second transfer occurs, these modes should undergo a coordinate transformation and reappear as a coherent vibration in the DK* manifold. However, when we simulate the DK* CVS originating from the transformed low-frequency modes of the first proton-transfer step (purple line in Figure 4a), a clear discrepancy emerges because the simulation predicts a mode at 238 cm^–1^ that is absent in the experiment.

Because the 238 cm^–1^ mode has a period of approximately 140 fs and the ultrafast reaction is completed in less than 100 fs,^26,28^ classical dephasing cannot account for the disappearance of this mode. Considering the relative amplitudes expected from the simulation, the absence of this mode is also unlikely to arise from limited experimental sensitivity. In the experimental spectrum, the 871 cm^–1^ peak, whose amplitude is approximately eight times smaller than that of the 200 cm^–1^ peak, is clearly resolved (Figure 2c and Figure S4a). Therefore, if a 238 cm^–1^ oscillation with an amplitude on the order of one-fifth to unity of the 200 cm^–1^ peak, as indicated by the simulations in Figure 4a, was present, it should be experimentally detectable. Thus, the lack of coherent signatures corresponding to the first proton-transfer step indicates that the dynamics cannot be readily explained within a semiclassical two-step proton-transfer mechanism.

**Figure 4:**
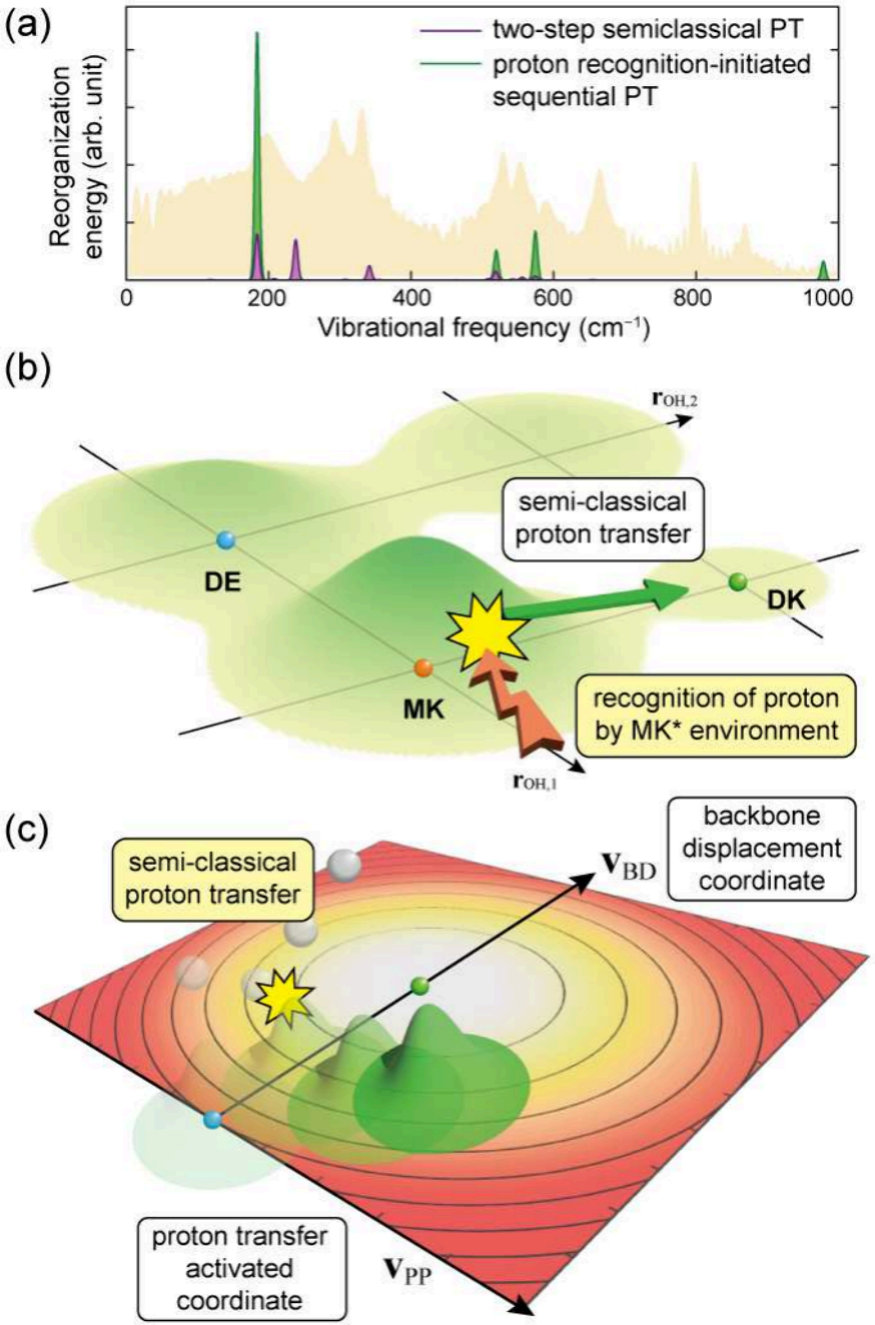
(a) CVS generated by the two-step semiclassical proton-transfer pathway (purple) and the proton-recognition-initiated sequential proton-transfer pathway (green). PT, proton transfer. (b) Schematic illustration of proton-position recognition immediately after photoexcitation. (c) Wavepacket generation along the backbone-displacement coordinate and the proton-transfer-activated coordinate.

Although the final product is identified as DK*, the observed vibrational coherence reflects only a single proton-transfer event (green traces in Figures 3b and 4a). This outcome implies that one of the two proton-transfer steps does not leave any detectable vibrational response that can be described within a semiclassical framework. The apparent contradiction can be resolved if we assume that the first proton is not recognized as having been transferred from the perspective of the molecule but is instead perceived as already residing near the acceptor. In other words, the system behaves as if the vibrational wave function of the asymmetric O–H stretch were distributed in the manner illustrated in Figure 1b. When this delocalized wave function interacts with the MK environment, the backbone responds as though the proton were already located at the acceptor (Figure 4b), and as a result no low-frequency vibrational coherence is produced from this step. It is important to emphasize that this does not imply a violation of momentum conservation. Rather, the key point is that the vibrational wave function along the proton-transfer coordinate does not evolve into a time-dependent wavepacket upon positional recognition.

Before proceeding further, it is important to consider the possible role of intramolecular vibrational redistribution (IVR). In the semiclassical picture, the formation of vibrational coherence following proton transfer can be viewed as a process in which energy initially deposited in high-frequency vibrational coordinates is redistributed among multiple modes, analogous to a sequence of IVR events. The modes appearing at 200 and 238 cm^–1^ in Figure 4a correspond to coordinates that can be activated by proton transfer and are therefore expected to possess stronger anharmonicity than typical backbone modes, making them more susceptible to subsequent IVR. Consistent with this picture, the 200 cm^–1^ peak generated by proton transfer exhibits a noticeably broader line width, indicating faster dephasing compared with other coherent vibrational modes. A similar tendency has been reported for the well-known ultrafast ESIPT molecule HBQ.^13–15^ Nevertheless, in both systems, the dephasing time remains on the order of several hundred femtoseconds, which is still sufficiently long to allow the observation of a coherent oscillation with a period of about 140 fs, corresponding to the 238 cm^–1^ mode.

A more direct correlation between vibrational coherence-based mechanistic insights and kinetic information would provide an additional understanding of the proton-transfer dynamics, particularly in systems where the proton transfer rate varies or where distinct wavepacket signatures are observed in the product states. However, as discussed above, TA measurements are significantly affected by coherent artifacts near time zero, which limit the reliability of the quantitative kinetic analysis. Experimental approaches that are less sensitive to such contributions, such as femtosecond time-resolved fluorescence^14^ or quantum optical femtosecond spectroscopy^43,44^ with pathway selectivity in the SE channel, are therefore expected to provide more quantitative insight into proton-transfer dynamics.

In an experiment detecting diffracted single photons, the position of the photon remains undetermined until it is detected on the screen. Although the photon’s initial position at the source is known, its location after emission is undefined until the measurement event collapses the probability distribution. A similar situation can be considered in the excited-state dynamics of BP­(OH)_2_. Before photoabsorption, both protons are localized at their donor sites. Immediately after excitation, however, the position of one of the two protons becomes uncertain and its location becomes defined only when the system interacts with its environment (Figure 4b). Both the DE and MK configurations can act as competing recognition channels, but MK* is more likely to serve as the outcome because its lower energy results in a higher density of vibrational states around the generated vibrational wave function, which in turn increases the probability that the proton is recognized in the MK* configuration. After this recognition event, the remaining proton-transfer step proceeds, ultimately yielding the DK* species. The coherent vibrational signatures observed in the experiment therefore arise from a combination of backbone structural displacements between the initial (GS) and final (DK*) states and the vibrational modes activated by the second proton transfer (Figure 4c).

In summary, the combined experimental and theoretical analyses reveal that the early time dynamics of BP­(OH)_2_ cannot be described by a purely semiclassical two-step proton-transfer mechanism. Instead, the data are consistent with a scenario in which one proton is effectively recognized in the MK* configuration immediately after excitation, leaving no detectable vibrational imprint from the first transfer. The subsequent proton transfer then proceeds semiclassically, generating the coherent vibrational features observed in the DK* state. These results uncover a quantum signature associated with proton recognition, a feature that is fundamentally absent in the semiclassical treatment of proton transfer. It is important to emphasize that this behavior should not be regarded as an exceptional phenomenon unique to this system. A closely related situation arises in photosynthetic energy-transfer systems, where localized excitons in the electronic eigenfunction regime rapidly evolve into states with different spatial probability density. Because nuclear motion is generally slower than electronic dynamics, proton transfer in condensed-phase chemistry is often treated within a semiclassical framework. The present results suggest that such an assumption may not always hold in ultrafast proton-transfer reactions and that coherent vibrational spectroscopy can reveal regimes in which nuclear dynamics exhibit analogous quantum behavior.

The pursuit of molecular systems with simple energy landscapes and long-lived coherence has become increasingly important, driven in part by their potential utility in emerging quantum technologies.^45^ While contributing to rapidly developing fields such as quantum information science is a valuable role for chemistry, it is equally essential for chemists to identify and investigate previously unrecognized phenomena that may shape future applications. In this context, studying molecular dynamics in regimes where semiclassical approximations break down is crucial, whether by employing quantum light to probe otherwise inaccessible interaction pathways^44,46,47^ or, as demonstrated in this work, by uncovering inherently quantum features embedded within processes traditionally described using semiclassical models.^16^

Our findings reveal that the early time dynamics of BP­(OH)_2_ involves a quantum signature associated with proton recognition that precedes and conditions the subsequent semiclassical proton-transfer step. This result demonstrates that even in condensed-phase photochemistry, where nuclear motion is commonly treated classically, quantum mechanical interpretation can be necessary to describe the underlying mechanism. Continued exploration of such quantum mechanical interpretation may not only refine our understanding of excited-state reactivity but also broaden the landscape of molecular phenomena relevant to future quantum-enabled technologies. Determining precisely what types of molecular systems exhibit such quantum-mechanical vibrational behavior will require further investigation, particularly through studies that combine ultrafast spectroscopy with rigorous quantum dynamical simulations.

## Supplementary Material



## Notes

Transient absorption spectroscopy, quantum chemical calculations, additional transient absorption data, normal mode projection, additional simulation results (PDF)
