# Supplementary material for: Quantum-Indeterminate Proton Positions in Ultrafast Excited-State Intramolecular Proton Transfer
Source: J Phys Chem Lett. 2026 Apr 29;17(19):5424–31. doi: 10.1021/acs.jpclett.6c01188 (PMC13181765; doi:10.1021/acs.jpclett.6c01188)
Supplement: Supplementary file 1 [file jz6c01188_si_001.pdf]

Supporting Information: Quantum-Indeterminate Proton Positions in Ultrafast Excited-State Intramolecular Proton Transfer

Minhyuk Lee[1], Changmin Lee*[2], and JunWoo Kim*[1]

[1]Department of Chemistry, Chungbuk National University, Cheongju 28644, Republic of Korea

[2]Department of Chemistry, Incheon National University, Incheon 22012, Republic of Korea

*Email: clee@inu.ac.kr, jwkim0@cbnu.ac.kr

Contents

S1 Transient Absorption Spectroscopy **S-2**

S1.1 Pump beam generation and pulse characterization S-2

S1.2 Probe beam generation S-2

S1.3 Data acquisition using a frequency-modulated rapid-scan method S-3

S2 Additional Transient Absorption Data **S-6**

S3 Quantum Chemical Calculations **S-7**

S4 Additional Analysis of Coherent Vibrational Spectra **S-8**

S5 Normal mode projection **S-9**

S6 Additional Simulation Results **S-11**

S6.1 Duschinski matrix S-11

S6.2 Additional simulation data S-12

S1 Transient Absorption Spectroscopy

S1.1 Pump beam generation and pulse characterization

The ultrafast transient absorption (TA) spectroscopy system was based on a Yb:KGW regenerative amplifier (Light Conversion, PHAROS-10W, 100 kHz, 100 μJ, 170 fs) as the fundamental light source. A home-built near infra-red (NIR) non-collinear optical parametric amplifier (NOPA) followed by second harmonic generation (SHG) provided the optical pump. The fundamental output was split into a 3 μJ beam and a 97 μJ beam, which were used to generate the seed and pump beams, respectively.

The 3 μJ beam was attenuated by a neutral density (ND) filter and focused into a 3 mm thick sapphire window (Eksma), generating a white-light supercontinuum (SC). A hard aperture was used to select the spatially uniform central portion (1 mm diameter) of the SC beam, and a harmonic separator (Eksma) removed the residual fundamental light. The resulting seed spanned 550—950 nm.

The 97 μJ beam was frequency doubled in a 2 mm-thick beta-barium borate (BBO) crystal (CASTECH, Type I, $\theta = 23.5°$) yielding 35 μJ pulses at 515 nm to pump the NOPA. A 515 nm harmonic separator (Eksma) removed the residual fundamental beam after SHG. The 515 nm pump and SC seed were focused into a 1.5 mm-thick BBO crystal (CASTECH, Type I, $\theta = 23.5°$) with focal lengths of 50 cm and 100 cm, respectively, and internal angle between the pump and the seed was set 2.3°. After amplification, the NOPA output spanned 650–950 nm, and the pulse energy was 2 μJ.

The amplified NIR-NOPA pulses were compressed using chirped mirror pairs (Layertec, 103366) and fused silica window pairs. Near-ultraviolet (NUV) pulses centered at 365 nm were generated via SHG in a 50 μm-thick BBO crystal (CASTECH, Type I, $\theta = 29.2°$). The NUV beam was separated from the residual NIR pulses using a harmonic separator (Layertec, 109433). Dispersion arising from air and optical elements was compensated using a pair of NUV chirped mirrors (Layertec, 148920) and a fused silica window (Eksma). The resulting NUV pulses serve as the pump pulse for the TA experiments.

Pulse characterization was performed using auto-correlation frequency-resolved optical gating (A-FROG) and cross-correlation frequency-resolved optical gating (X-FROG). We separated NIR pulses with 50:50 beam splitter (Layertec, 104028) as a gate pulse before the frequency doubling. The gate and the probe pulses (NIR, NUV both) were focused into a 10 μm thin BBO crystal (CASTECH, Type I, $\theta = 29.2°$ for NIR pulses and $\theta = 44.3°$ for NUV pulses) at a sample position. Delay between gate and probe pulse adjusted by a motorized stage (Newport, XMS160) driven by a motion controller (Newport, XPS-RLD4). The resulting pulse duration of the NIR and NUV pulse were estimated as 10 fs and 15 fs, respectively (Figure S1).

S1.2 Probe beam generation

Two probe beams were generated to cover different detection windows for the stimulated emission (SE) and excited state absorption (ESA) bands. Both probes were derived from

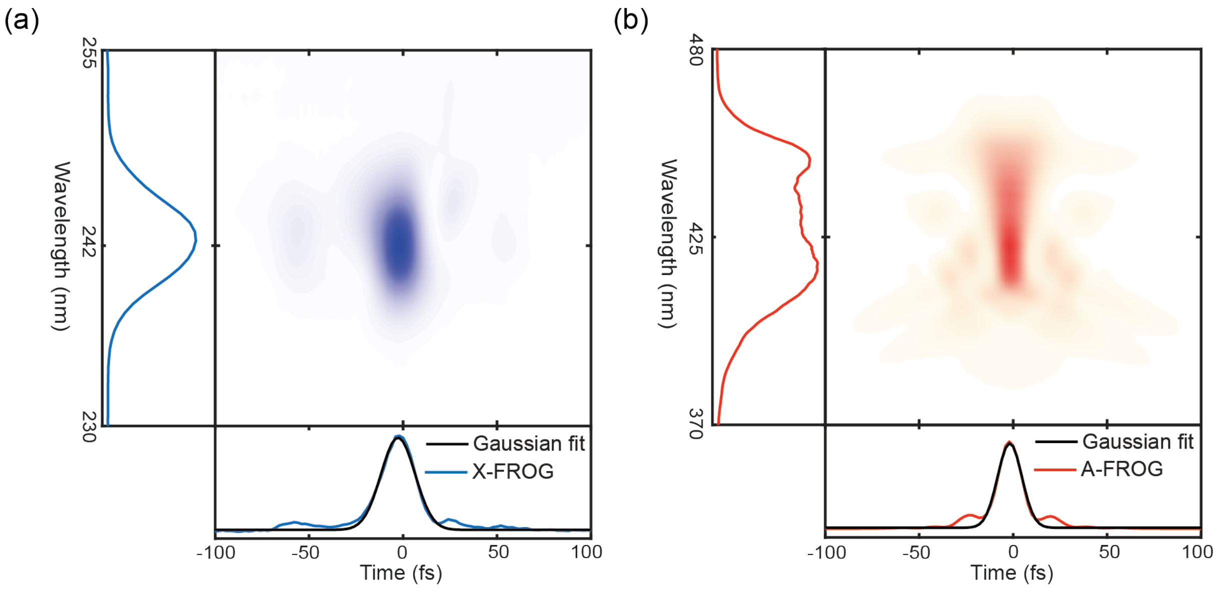

Figure S1: Pulse characterization of the TA pump and NOPA output. (a) X-FROG trace of the pump pulse used for TA; the retrieved temporal profile gives an FWHM of 20 fs (Gaussian fit). (b) A-FROG trace of the NOPA output; the temporal FWHM is 14 fs (Gaussian fit).

the residual fundamental output remaining after the NOPA. Although a single broadband white-light continuum can in principle be generated using a CaF_2 plate, we found that achieving sufficient long-term stability under our experimental conditions was challenging. Therefore, two independent white-light continua were generated and used separately in the measurements.

For detection of the SE band, a 2 μJ beam picked off from the fundamental output was focused into a 3 mm thick sapphire window (Eksma) using optics with a focal length of 10 cm, generating a SC spanning 480850 nm. The spatially uniform central portion of the SC (1 mm diameter) was isolated using a hard aperture and used as the probe for SE detection.

For detection of the ESA band, the fundamental beam was frequency-doubled in a 2 mm thick BBO crystal (CASTECH, Type I, $\theta = 23.5°$) to generate a 515 nm beam with a pulse energy of 3 μJ. The 515 nm beam was focused into a 3 mm thick sapphire window (Eksma) using optics with a focal length of 10 cm, producing an SC spanning 380–480 nm. The probe beam for ESA detection was defined by aperturing the spectrally uniform central region of the supercontinuum (1 mm diameter).

S1.3 Data acquisition using a frequency-modulated rapid-scan method

The sample solution was prepared by dissolving 2,2'-bipyridine-3,3'-diol (TCI, >99.8%) in cyclohexane (DAEJUNG, HPLC grade, >99.8%) to a concentration of 2 mM. Steady-state absorption and fluorescence spectra were recorded prior to the transient absorption measurements. The absorption maximum appeared at 341 nm in acetonitrile and 345 nm in cyclohexane, and the pump spectrum was located within the absorption band of the

sample (Figure S2). The sample was circulated through a 200 μm path-length fused silica flow cell using a gear pump. The pump pulse energy at the sample was 20 nJ with a beam waist diameter of $\tilde{2}00$ μm, while the probe beam waist diameter was $\tilde{1}00$ μm. The probe beam transmitted through the sample was collected and focused into an optical fiber coupled to a spectrometer (Avantes, AvaSpec-ULS2048CL-EVO) equipped with a CMOS array detector. The pumpprobe delay was controlled by a motorized delay stage (Newport, XMS160) driven by an XPS motion controller (Newport, XPS-RLD4).

The rapid-scan TA system operates by continuously scanning the delay stage at a constant velocity to measure pump-on and pump-off data as a function of delay time. To ensure precise timing, all instruments—including the light source, spectrometer, and optical chopper—were synchronized using a function generator (Keysight, EDU33212a) as a master clock. The master clock generated two signals corresponding to the repetition frequency of the light source, f_{rep} (100 kHz) and the readout frequency, f_{RO} (2 kHz) of the spectrometer, respectively.

An optical chopper (Thorlabs, MC1F10HP blade with MC2000B-EC driver) was driven at 1 kHz by dividing f_{RO} by two. The spectrometer was synchronized to f_{RO} to capture spectra for alternating pump on/off states at each point. The position of the delay stage was recorded by triggering the motion controller with the reference output signal from the optical chopper during the pump on state. If scanning speed is too fast, the time delay difference between the pump on and pump off state may exceed the temporal resolution of the setup. To prevent this problem, velocity of the stage was set to 150 μm/s, resulting in a time interval of 1 fs. Spectra acquired at 2 kHz were initially stored in an on-board memory of the spectrometer and transferred to a PC after each scan. For all measurements, the TA spectra were averaged over 2000 scans.

The raw pump-on/off spectra were processed based on the recorded stage positions. To generate a uniform time axis, the data were interpolated and subsequently smoothed by convolution with a Gaussian filter having a temporal width of 10 fs, yielding TA spectra with a 10 fs time step. These spectra were averaged in blocks of 50 scans. Time-zero correction was applied to each block to correct for the group velocity dispersion (GVD) of the broadband SC probe, using the coherent artifact as a reference. Additionally, drifts in time-zero that occurred over the measurement duration were corrected. Finally, to obtain a coherent vibrational spectrum (CVS), the TA spectra were analyzed by fitting to a decay model, and the residuals were Fourier transformed.

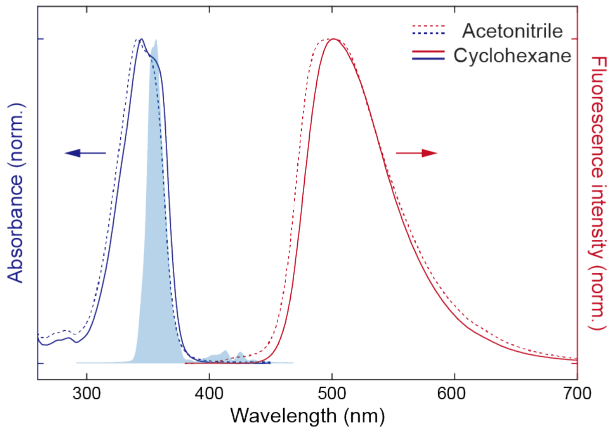

Figure S2: Normalized steady-state absorption (blue) and fluorescence emission (red) spectra of $BP(OH)_2$ measured in acetonitrile (dashed lines) and cyclohexane (solid lines). The shaded blue profile denotes the pump spectrum employed for the pumpprobe experiments. The pump spectrum overlaps with the absorption band in both solvents.

S2 Additional Transient Absorption Data

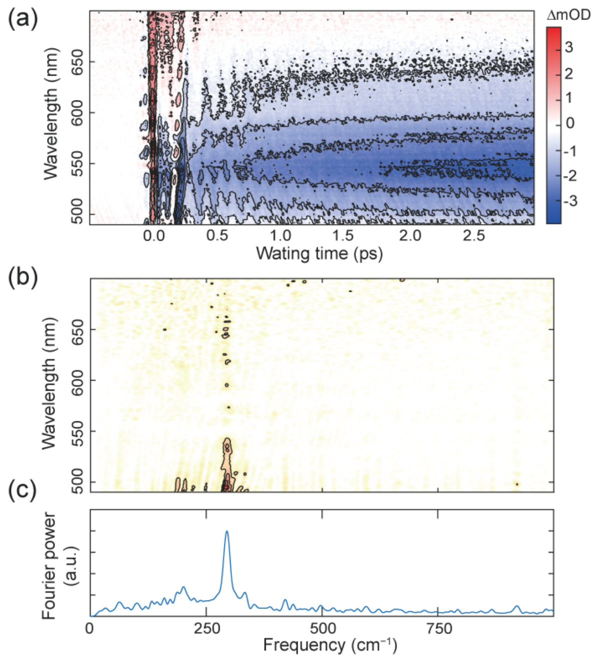

Figure S3: (a) TA spectrum of $BP(OH)_2$ dissolved in acetonitrile. Positive and negative signals are shown in red and blue, respectively. (b) Coherent vibrational map obtained from the TA data in panel a. (c) Coherent vibrational spectra extracted from the red side of the SE band.

S3 Quantum Chemical Calculations

All electronic structure calculations were performed using the Gaussian 16 software package.[1] To accurately describe the initial enol form (GS), excited mono-keto (MK*), and excited di-keto (DK*), we employed density functional theory (DFT) and time-dependent DFT (TD-DFT) for the ground and excited states, respectively. The CAM-B3LYP functional was used for all calculations, as it incorporates long-range corrections, which are essential for providing a reliable description of excited-state geometries in ESIPT systems.[2] The 6-31G(d,p) basis set was used to balance computational cost with the precision required for vibrational analysis, and all calculations were done in the gas phase. The vibrational frequencies of the MK* and DK* states exhibit clear differences across corresponding normal modes, as shown in Figure S4.

Equilibrium geometries for the ground state and the first singlet excited state were optimized using standard convergence criteria. The singlet excited state was confirmed to be the optically accessed state in the experiment, as evidenced by its oscillator strength of appropriate magnitude. Harmonic vibrational frequency calculations were performed at the same level of theory to ensure that all the optimized structures correspond to true local minima. The calculated frequencies for MK* and DK* were used to simulate the CVS and to identify the normal modes coupled to the ESIPT reactions, as discussed in the main text. The detailed methodology for the normal mode projection procedure is described in the Supporting Information Section 5: Normal mode projection.

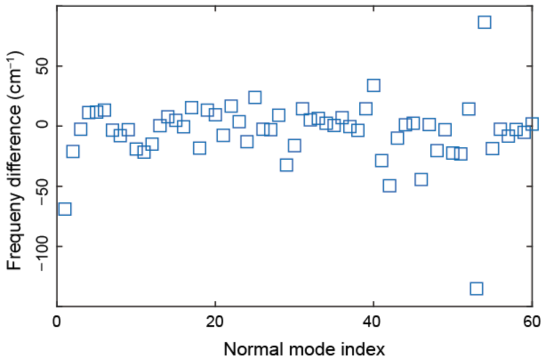

Figure S4: Vibrational frequency differences between corresponding normal modes of the excited mono-keto (MK*) and di-keto (DK*) forms.

S4 Additional Analysis of Coherent Vibrational Spectra

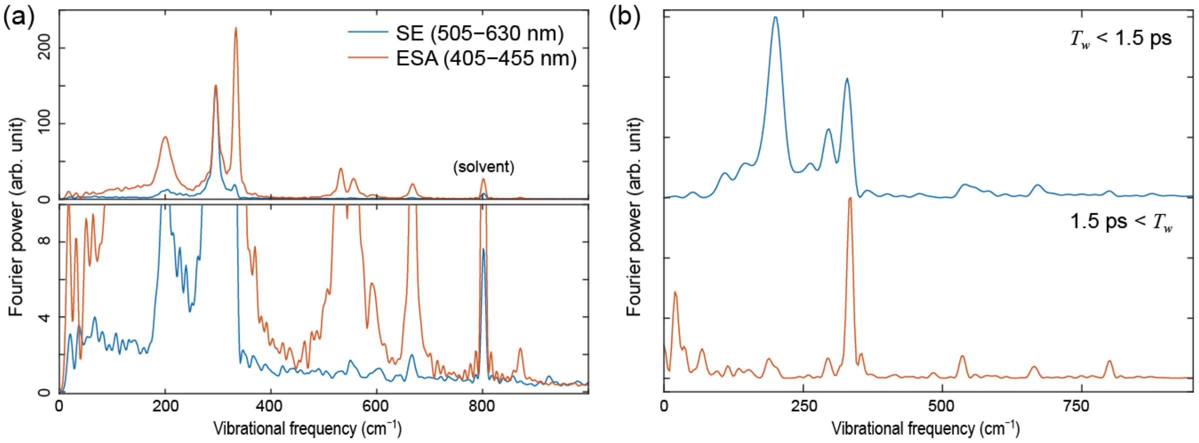

Figure S5: (a) Linearly scaled coherent vibrational spectra extracted from the stimulated emission (blue) and excited-state absorption (orange) regions. The lower panel shows a magnified view of the upper panel. (b) Coherent vibrational spectra obtained using two time windows, T_w <1.5 ps (top, blue) and T_w >1.5 ps (bottom, orange).

S5 Normal mode projection

Electronic transitions between two electronic states are accompanied by excitation of vibrational modes that are vibronically coupled to the transition. The extent of this excitation is determined by the vibronic coupling strength for each normal mode, denoted as λ_i, where i indicates the vibrational modes. These mode-specific coupling strengths are also referred to as vibrational reorganization energies and, within a semiclassical picture, are governed by the vibrational displacements, δ_i, between the equilibrium geometries of the two electronic states. Because the amplitudes of vibrational wavepackets observed in femtosecond spectroscopic experiments are directly related to λ_i, wavepacket analysis provides a means of extracting information about the structural differences between the initial electronic state and the transient equilibrium geometry following excitation.

In terms of the curvilinear coordinates, the displacement of ith mode, δ_i, with respect to the structural difference between the equilibrium geometries of the initial (denoted as *ini*) and final (denoted as *fin*) electronic states, $\Delta\mathbf{q}_C \equiv \mathbf{q}_{C,fin} - \mathbf{q}_{C,ini}$, where the ith vibrational mode belongs to the final state, is given by

$$\boldsymbol{\delta} = -\mathbf{l}^{-1}\mathbf{c}^T\mathbf{m}^{-1/2}\mathbf{B}^{-1}\Delta\mathbf{q}_C, \tag{S1}$$

$\mathbf{m}$ is the diagonal mass matrix, whose non-zero elements are the atomic masses associated with each Cartesian coordinate. $\mathbf{c}$ is the matrix whose columns are the mass-weighted normal-mode eigenvectors. It transforms mass-weighted Cartesian displacements into normal-coordinate displacements. $\mathbf{B}$ is Wilsons B matrix, which relates Cartesian displacements to changes in internal coordinates. Lastly, $\mathbf{l}$ is a diagonal matrix whose elements are the zero-point amplitudes of each normal mode,

$$l_{ii} = \left(\frac{\hbar}{2\pi\nu_i}\right)^{1/2}, \tag{S2}$$

The projections also provide the reorganization energies associated with states 1 and 2. Within the harmonic approximation, the total reorganization energies may be expressed as

$$\boldsymbol{\lambda} = \sum_{i=1}^{N_v} \lambda_i, \qquad \lambda_i = \frac{1}{2}h\nu_i(\delta_i)^2, \tag{S3}$$

These mode-resolved contributions quantify the coupling between nuclear and electronic motions and also allow direct evaluation of vibronic coupling constants. The normal-mode projection procedure based on curvilinear coordinates is described in detail in paper[3], and has been widely employed for analyzing excited-state geometries[4] as well as ultrafast reaction dynamics.[5]

The orange curves in Figures 3a and 3b represent CVS signals reconstructed from the displacements ($\boldsymbol{\delta}$) between the ground-state enol-form structures and the excited-state minima. Specifically, two modified ground-state geometries were considered: one in which both protons were repositioned (GS_2) and another in which only one proton

was repositioned (GS_1). For each case, the final-state geometriesthe excited diketo-form (DK*) for GS_2 and the excited monoketo-form (MK*) for GS_1were used as references to compute the displacements along the normal coordinates of the excited-state minima (DK* and MK*).

The protons in GS_1 and GS_2 were displaced so that their positions reproduced the corresponding protonacceptor distances and the protonacceptorcarbon angles found in the DK* and MK* optimized structures. This procedure isolates the geometric contribution arising from backbone reorganization while excluding large nonlinearities associated with full proton transfer. It also enables the proton-transfer-induced contributions to the formation of the vibrational wavepacket to be treated separately in the simulations.

S6 Additional Simulation Results

S6.1 Duschinski matrix

Ultrafast reactions that involve substantial structural rearrangements, such as excited-state intramolecular proton transfer (ESIPT), make it challenging to identify vibrational coherences in the product state. If a vibrational mode initially activated in the reactant (denoted as *ini*) is not strongly involved in the reaction pathway, its coherence is largely transferred to a single normal mode of the product (denoted as *fin*). However, when a particular vibrational mode contributes significantly to the structural change along the reaction coordinate, the corresponding coherence is redistributed among multiple normal modes in the product state. The redistribution coefficients can be defined by expressing the motion of the initial vibrational mode as a linear combination of the normal modes in the product. By applying the transformation between curvilinear coordinates and normal modes described in Supporting Information Section 4, it becomes possible to construct the transformation matrix, $\mathbf{D}_C$, that connects these two sets of vibrational modes, as shown below.

$$\mathbf{D}_C = \mathbf{c}_{ini}^{\prime T} \mathbf{G}_{ini}^{\prime -1/2} \mathbf{a}_{ini}^T \mathbf{B}_{fin} \mathbf{m}^{-1/2} \mathbf{c}_{fin}. \tag{S4}$$

where

$$\mathbf{c}_i'' = \mathbf{B}_i'' \mathbf{m}^{-1/2} \mathbf{c}_i, \tag{S5}$$

$$\mathbf{B}_i'' = \mathbf{G}_i^{\prime -1/2} \mathbf{a}_i^T \mathbf{B}_i. \tag{S6}$$

and

$$\mathbf{G}_i' = \mathbf{B}_i' \mathbf{m}^{-1} \mathbf{B}_i^{\prime T}, \tag{S7}$$

$\mathbf{a}_i$ is the transformation matrix that connects the curvilinear internal coordinates of the ith state with the normal vibrational modes.

Although the transformation between vibrational modes and curvilinear internal coordinates relies on the assumption that the structural changes involved are not excessively large, in practice, the squared sum of the transformation coefficients does not necessarily equal unity. Even so, the resulting Duschinsky matrix remains highly useful for analyzing structural changes between different electronic states. It is important to note that the coherence amplitude observed in the product state depends not only on the magnitude of the transformation coefficients, but also sensitively on the frequency differences and relative phases of the coupled vibrational modes. While an analytical treatment of these contributions is generally difficult, numerical simulations allow reliable prediction of the vibrational coherences that appear in the product for systems in which ESIPT proceeds through a single transfer event.

S6.2 Additional simulation data

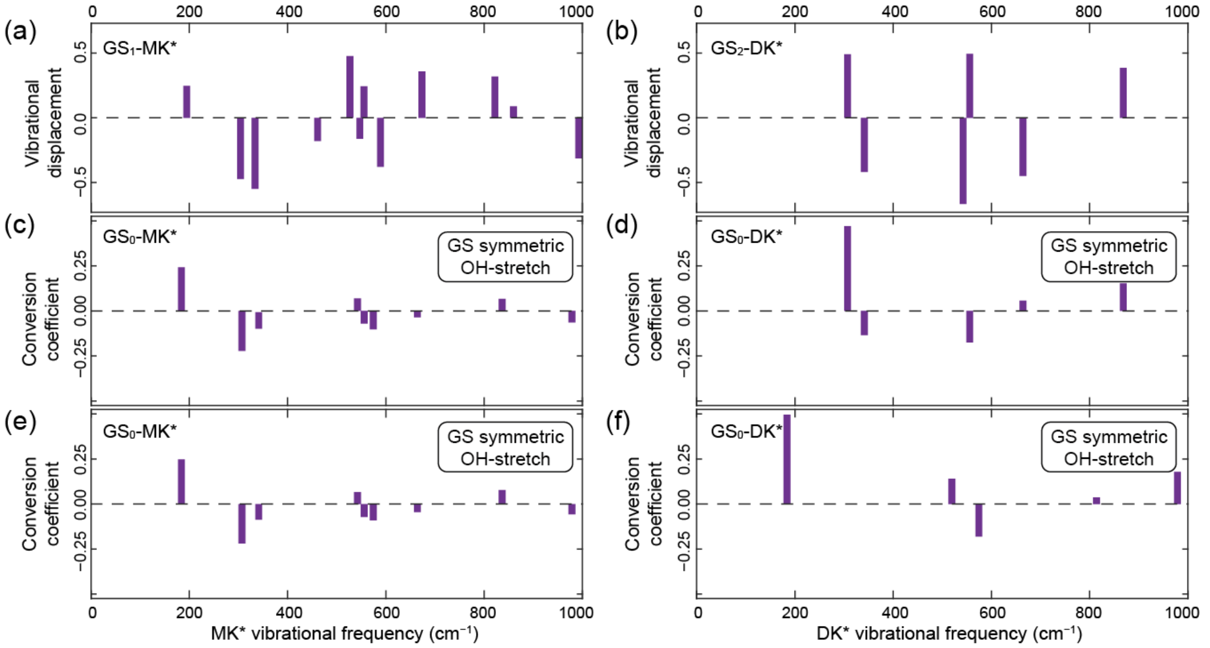

Figure S6: (a-b) Vibrational displacements of the normal modes of MK* (a) and DK* (b) obtained from projecting the GS optimized structure onto the corresponding excited-state normal modes. (c-d) Duschinsky matrix elements of MK* (c) and DK* (d) associated with the GS symmetric OH stretch mode. (e-f) Duschinsky matrix elements of MK* (e) and DK* (f) associated with the GS asymmetric OH stretch mode.

References

(1) Frisch, M. J. et al. Gaussian 16 Revision C.01, Gaussian Inc. Wallingford CT, 2016.

(2) Yanai, T.; Tew, D. P.; Handy, N. C. A new hybrid exchange-correlation functional using the Coulomb-attenuating method (CAM-B3LYP). *Chem. Phys. Lett.* **2004**, *393*, 51–57.

(3) Reimers, J. R. A practical method for the use of curvilinear coordinates in calculations of normal-mode-projected displacements and Duschinsky rotation matrices for large molecules. *J. Chem. Phys.* **2001**, *115*, 9103–9109.

(4) Kim, J.; Kim, D. E.; Joo, T. Excited-State Dynamics of Thioflavin T: Planar Stable Intermediate Revealed by Nuclear Wave Packet Spectroscopies. *J. Phys. Chem. A* **2018**, *122*, 1283–1290.

(5) Kim, J.; Kim, C. H.; Burger, C.; Park, M.; Kling, M. F.; Kim, D. E.; Joo, T. Non-Born–Oppenheimer molecular dynamics observed by coherent nuclear wave packets. *J. Phys. Chem. Lett.* **2020**, *11*, 755–761.
